# Use of analgesics in France, following dextropropoxyphene withdrawal

**DOI:** 10.1186/s12913-018-3058-1

**Published:** 2018-04-02

**Authors:** E. Van Ganse, M. Belhassen, M. Ginoux, E. Chrétien, C. Cornu, C. Ecoffey, F. Aubrun

**Affiliations:** 1 HESPER 7425, Health Services and Performance Research, University Claude Bernard Lyon 1, Lyon, France; 2 PELyon, PharmacoEpidemiologie Lyon, Lyon, France; 3 Respiratory Medicine, Croix Rousse University Hospital, Lyon, France; 4 Department of Anesthesiology and Critical Care, Croix Rousse University Hospital, Claude Bernard Lyon 1 University, Lyon, France; 5 UMR 5558, Laboratoire de Biométrie et Biologie Evolutive, Claude Bernard University, CNRS, Lyon, France; 6 Louis Pradel Hospital, Lyon University, Lyon, France INSERM Clinical Investigation Centre (CIC1407), Lyon, France; 7 Department of Anaesthesiology and Critical Care, Ponchaillou University Hospital, CIC Inserm, Rennes, France

**Keywords:** Pain management, Drug withdrawal, Real-life use, Analgesics

## Abstract

**Background:**

In 2009, the European Medicines Agency recommended withdrawal of dextropropoxyphene (DXP); in March 2011 it was withdrawn from the market in France. Up until that time the combination dextropropoxyphene-paracetamol (DXP/PC) was widely used for analgesia. At withdrawal, French regulators recommended that DXP/PC be replaced by other step 2 analgesics, i.e. tramadol, codeine, or opium-containing drugs, or by PC for a weak level of pain. To investigate prescribing behaviours after DXP/PC withdrawal, dispensations of analgesics before and after withdrawal were analysed.

**Methods:**

Aggregated dispensation data of analgesics prescribed between January 2009 and December 2012 in the Rhône-Alpes region were obtained from the general health insurance claims data; changes in analgesic dispensation over time were analysed with the ATC/DDD methodology. Pre (Jan-June 2009) and post-withdrawal (Jan-June 2012) changes of DDDs where computed for each analgesic step.

**Results:**

The dispensations of DXP/PC experienced a two-step decrease until 2011. Over the withdrawal period 2009-2012, there was a 14% decrease in the overall use of analgesic (from 109 to 94 DDDs), while the use of step 2 analgesics declined by 46% (− 22 DDDs, from 47 to 25 DDDs). This latter decline included a cessation of use of DXP/PC (29 DDDs in 2009) that were only in part (+ 7 DDDs, from 18 to 25 DDDs) compensated by increased use of codeine, tramadol and opium, in monotherapy or combined with PC. For step 1 analgesics, use increased with 9%, mostly PC (+ 8 DDDs, from 31 to 39 DDDs). Step 3 analgesics dispensations remained largely unchanged over this period (around 3 DDDs).

**Conclusions:**

In the Rhône-Alpes region, DXP/PC withdrawal was accompanied in part by an increased use of same level analgesics, and in part by an increased use of PC in monotherapy. The extent of DXP/PC use before withdrawal, and the increased use of PC after DXP withdrawal, underline the complexity of pain management.

## Background

A rational approach to the treatment of pain is to combine treatments that act on distinct pain mechanisms in order to improve analgesia and, hopefully, to reduce the incidence of adverse events [1]. This is the concept of multimodal analgesia. The World Health Organization (WHO) three-step Analgesic Ladder, proposed for cancer pain in 1986, is a stepwise approach to analgesic management, where a patient’s pain severity determines the level of analgesics [2]. In this ladder, when pain is not relieved by WHO step 1 analgesics such as paracetamol (PC) or non-steroidal anti-inflammatory drugs (NSAIDs), a WHO step 2 analgesic, i.e. one of three weak opioids – codeine, tramadol or dextropropoxyphene (DXP) – is recommended, generally in combination with PC.

However, DXP-related hepatotoxicity and its frequent use for suicidal poisoning reported in North Europe, United States and Australia, led to its progressive withdrawal. For instance, in the United Kingdom (UK), co-proxamol was withdrawn in 2007 [3]. In 2009, the European Medicines Agency (EMA) recommended the withdrawal of DXP/PC throughout the European Union [4]. Two years later, and despite objections from the French health authorities based on the benefit/risk ratio considered to be locally acceptable, DXP/PC was totally withdrawn from the French market, in March 2011 [5].

DXP/PC was widely used in France with more than 70 million of DXP/PC packs sold per year [6]. Until market withdrawal in France, DXP/PC was the second most prescribed analgesic drug after PC [7], and there were 41 different medications containing DXP/PC.

To accompany the withdrawal, the French Regulators, in collaboration with scientific societies, provided recommendations regarding therapeutic alternatives to DXP/PC. It was suggested to replace DXP/PC by another step 2 analgesic, e.g. tramadol or codeine, and by PC for weaker pain level [8].

While the consequences of DXP/PC withdrawal on suicidal deaths have been studied [9–15], its effect on overall analgesic prescriptions have been little investigated [3]. Due to their specific benefit/risk ratios and their extensive use, drugs replacing DXP/PC have impacted the management of pain, and the quality of care. These changes need to be detailed, first of all in terms of use of analgesics.

To provide a first set of data, an analysis of the Rhône-Alpes URCAM (Regional Union of Health Insurance Fund) database with 5 million inhabitants covered by the general health insurance scheme was performed, to describe analgesics dispensation between 2009 and 2012, around the time of DXP/PC withdrawal.

## Methods

### Analysis of analgesic dispensations

To investigate DXP/PC replacement, dispensation data of all prescribed analgesics were requested for the period January 2009 to December 2012 for the Rhône-Alpes region of France. The regional fund that is part of the national health insurance system [16] provided a dataset containing records of all analgesics that had been dispensed during that period to patients covered by the general health insurance scheme (i.e. 80% of the regional population). This extraction was based on CIP code (specific national identification code of medicinal products) of all analgesics (Table 1).

**Table 1:** Selected therapeutic classes or medications and corresponding analgesic steps

Analgesics Selection In The Urcam Rhone-Alpes Reimbursement Database		
Step 1	Step 2	Step 3
Non Steroidal Anti-Inflammatory Drugs (NSAIDs)	Codeine	Morphine-Like Drugs
Paracetamol (PC)	Dextropropoxyphene	Morphine
	Tramadol	
	Paracetamol + Codeine	
	Paracetamol + Opium	
	Paracetamol+Tramadol	

The aggregated number of packs monthly dispensed was computed (in France drugs are sold as indivisible units). For the analyses, the Anatomical Therapeutic Chemical classification/defined daily dose (ATC/DDD) methodology of the WHO Collaborating Centre for Drug Statistics Methodology was used [17]. The dispensation of oral analgesics was described over time by DDDs/1000 inhabitants/day.

The changes of DDDs between January-June 2009 and January-June 2012 for each analgesic step were computed by subtracting the numbers of DDDs/1000 inhabitants/day of each medicinal product reimbursed during the pre-withdrawal period (Jan-June 2009) from the numbers of DDDs reimbursed during the post-withdrawal period (Jan-June 2012), divided by the total numbers of DDDs reimbursed during the pre-withdrawal period.

All analyses were performed using SAS software, version 9.4 (SAS Institute Inc., Cary, NC, USA).

## Results

### DXP/PC use over the period 2009-2012

A total of 36 different DXP-containing medications were identified. In 2009, the most common DXP-containing medication was a fixed combination containing 30 mg DXP, and 400 mg PC (DXP 27MG/PC 400 MG). This combination represented 69.6% of all dispensations of DXP-containing medications. All profiles of use evolved similarly, with a two-step decrease of DXP/PC between 2009 and 2012. A first decrease occurred in June 2009, following an EMA announcement of DXP/PC withdrawal from the European market. A second, and final, decrease occurred in March 2011 at the time of withdrawal in France.

### Parallel use of other analgesics

Between 2009 and 2012, the total use of analgesics - expressed in DDDs - decreased with 14% (Table 2).

**Table 2:** Number of DDDs/1000 inhabitants/day of analgesics during the pre-withdrawal period (Jan-June 2009) and the post-withdrawal period (Jan-June 2012)

	Pre-withdrawal	Post-withdrawal	Percentage changes
WHO ladder step 1			
NSAIDs	29,41	26,53	−9,8%
PC	30,79	39,16	+ 27,2%
ALL STEP 1	60,20	65,69	+ 9,1%
WHO ladder step 2			
DXP / PC	28,82	0,00	−100%
CODEINE	0,03	0,05	+ 50,0%
CODEINE / PC	3,04	5,06	+ 66,3%
OPIUM / PC	1,40	2,55	+ 83,0%
TRAMADOL	3,58	3,99	+ 11,3%
TRAMADOL / PC	9,68	13,39	+ 38,3%
ALL STEP 2	46,56	25,05	−46,2%
WHO ladder step 3			
MORPHINE-LIKE DRUGS	1,90	2,50	+ 31,3%
MORPHINE	0,79	0,84	+ 5,5%
ALL STEP 3	2,70	3,33	+ 23,7%
ALL ANALGESICS	109,45	94,07	−14,1%

**Fig. 1:**
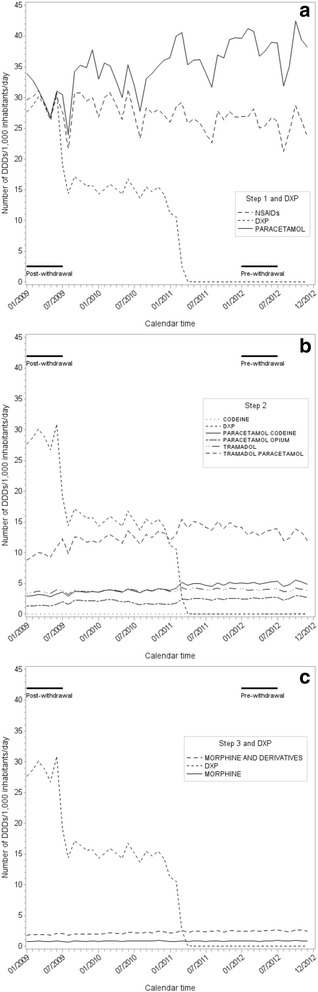
Use of step 1, step 2, and step 3 analgesics over the period 2009-2012, in DDDs/1000 inhabitants/day

Over the same period, the total number of DDDs of step 2 analgesics declined by 46% (from 47 to 25 DDDs, Fig. 1). This was mostly due to the withdrawal of DXP/PC (29 DDDs) in 2011 in parallel to an increased use of step-2 analgesics (codeine, opium, and tramadol, mostly combined to PC: increase of 7 DDDs). For step 1 analgesics, there was an increase of 5 DDDs (+ 9%), due to PC (Fig. 1). Step 3 analgesics dispensations increased with 24% over this period, but their use remained limited (3 DDDs in 2012, Fig. 1).

**Fig. 2:**
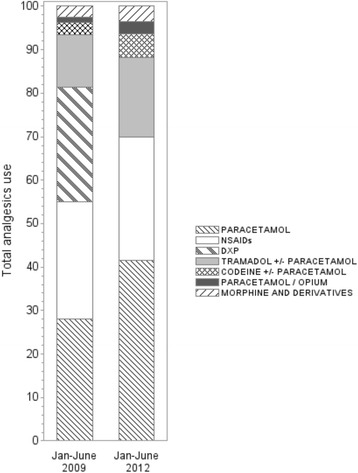
Percentages of treatments among total analgesic use in Jan-June 2009 and Jan-June 2012

The distribution of use of analgesics during the first six months (Jan-June) of 2009 and the first six months of 2012 (Fig. 2) shows the increased proportion of PC among analgesic use.

## Discussion

In France, the withdrawal of DXP/PC took place in two phases, with a first decrease in 2009 following an EMA opinion, and a second, final decrease in 2011 due to national regulatory decisions. The global dispensation data of prescribed analgesics suggest that the DXP/PC withdrawal had a small impact on the overall use of analgesics in France, as the total dispensations of these drugs decreased by 14% over the four years considered. However, over the same period, there was an increased use of PC, a step 1 analgesic, and to a lesser extent, of step 2 analgesics, codeine, opium, and tramadol, mostly combined with PC.

The data suggest that physicians replaced DXP/PC quickly after withdrawal from the French market. This was not straightforward, as this medication had been widely prescribed since 1964 – e.g., figures from 2009 show a use of 29 DDDs/1000 inhabitants/day – in a large set of indications. Part of the explanation for this quick replacement could be the fact that the benefit-risk ratio of DXP/PC was considered – by EMA, health care professionals (HCPs) and scientific societies – to be disputable. For instance, in 2008, a consensus conference on postoperative pain care concluded that DXP/PC should not be prescribed for this indication [18]. Surveys conducted among HCPs also revealed concerns regarding the safety profile or the limited efficacy of DXP/PC [19].

However, the use of analgesics after DXP withdrawal had not been predicted. Before withdrawal, step 2 analgesics, particularly tramadol/PC and codeine/PC, were expected to be used much more frequently, but our data show that after DXP withdrawal, the use of PC increased more than the use of step 2 analgesics. The reasons for this limited increase of step 2 analgesics could be related to safety concerns as tramadol is known for its poor tolerability, while codeine is under surveillance for its respiratory effects [20–22]. Also, opium-containing drug are seldom prescribed, except for elderly patients. In that context, for the prior indications of DXP/PC, prescribers probably chose PC, i.e. a less effective, but safe alternative to step 2 analgesics.

Of interest, the results of this study differ from the results of a survey performed among Pain specialists asked to describe alternatives to DXP in France [23]. HCPs declared tramadol combined with PC to be the substitutive analgesic of choice, while only 24% of considered PC alone as a substitute.

By contrast, another study conducted in a teaching hospital in 1997, i.e. long before withdrawal, suggested that DXP/PC should be predominantly replaced by PC alone, in agreement with our findings [7]. Also in line with our data, a study conducted after withdrawal among community-dwelling elderly suffering from chronic pain and previously treated with DXP/PC, showed that a majority of patients remained treated with step 2 analgesics, mainly tramadol, but that 40% were switched to step 1 drugs [24]. Altogether, the available data suggest that the choice of replacement analgesics depended on physician specialty, setting – e.g. primary vs. secondary care – indication, patients’ comorbidities and age.

The effects of DXP withdrawal have also been investigated in other countries, notably in the UK, where withdrawal was effective in 2008. In the UK, a 23%-increase in codeine/PC, a 19%-increase in tramadol and a 16%-increase in PC prescriptions were reported [25], confirming international differences in pain management.

Our findings had some limitations. This study relied on the use of aggregated data, i.e. monthly dispensations delivered to a population of five million people after analgesic prescribing by regional physicians. As such, it was not possible to distinguish successive episodes of use of analgesics in individuals, to identify analgesic therapy prescribed after DXP/PC withdrawal in chronic or repeated users. It was also not possible to assess the impact of therapeutic changes on the effectiveness of pain therapy, in the absence of patient-reported data. Access to individual drug histories would have allowed exploring differences in patients’ characteristics, such as gender, age or comorbidities, and differences in prescribers’ specialties, in addition to providing some markers of treatment effectiveness.

Also, our data did not allow to verify the occurrence of a storage phenomenon that was shown to delay DXP replacement in the UK, where 30% of patients were still using DXP/PC one year following its withdrawal [25].

A last limitation refers to the absence of over-the-counter data, since claims data include only information on drugs that were both prescribed and dispensed. However, prior research on this issue support the validity of the results obtained with claims data, as in France, PC is mostly used as prescribed therapy, while step 2 and step 3 analgesics are Prescription-Only-Medicines [26].

## Conclusion

In conclusion, after DXP withdrawal, large increases in PC use were observed, while the use of other step 2 analgesics increased to a lesser extent. Detailed analyses of individual longitudinal drug histories would be helpful to confirm the nature and the effectiveness of replacement therapy according to patients’ and prescribers’ characteristics, and the kinetics of this process, besides allowing investigations of quality of care in analgesia, and much-needed international comparisons.

## Abbreviations

ATC: Anatomical Therapeutic Chemical
CAF: Caffeine
DDD: Defined Daily Dose
DXP: Dextropropoxyphene
DXP/PC: Dextropropoxyphene/Paracetamol
EMA: European Medicines Agency
HCP: Health Care Professionals
NSAIDs: Non-Steroidal Anti-Inflammatory Drugs
PC: Paracetamol
UK: United Kingdom
URCAM: Regional Union of Health Insurance Fund
WHO: World Health Organization
